# Muscle synergy analysis of eight inter-limb coordination modes during human hands-knees crawling movement

**DOI:** 10.3389/fnins.2023.1135646

**Published:** 2023-05-19

**Authors:** Chengxiang Li, Xiang Chen, Xu Zhang, Xun Chen, De Wu

**Affiliations:** ^1^School of Information Science and Technology, University of Science and Technology of China, Hefei, Anhui, China; ^2^Department of Pediatrics, The First Affiliated Hospital of Anhui Medical University, Hefei, China

**Keywords:** surface electromyography, hands-knees crawling, inter-limb coordination, muscle synergy, neuromuscular control mechanism

## Abstract

In order to reveal in-depth the neuromuscular control mechanism of human crawling, this study carries out muscle synergy extraction and analysis on human hands-knees crawling under eight specific inter-limb coordination modes, which are defined according to the swing sequence of limbs and includes two-limb swing crawling modes and six single-limb swing crawling modes. Ten healthy adults participate in crawling data collection, and surface electromyography (sEMG) signals are recorded from 30 muscles of limbs and trunk. Non-negative matrix factorization (NNMF) algorithm is adopted for muscle synergy extraction, and a three-step muscle synergy analysis scheme is implemented by using the hierarchical clustering method. Based on results of muscle synergy extraction, 4 to 7 synergies are extracted from each participant in each inter-limb coordination mode, which supports the muscle synergy hypothesis to some extent, namely, central nervous system (CNS) controls the inter-limb coordination modes during crawling movement by recruiting a certain amount of muscle synergies, rather than a single muscle. In addition, when different participants crawl in the same inter-limb coordination mode, they share more temporal features in recruiting muscle synergies. Further, by extracting and analyzing intra-mode shared synergies among participants and inter-mode shared synergies among the eight inter-limb coordination modes, the CNS is found to realize single-limb swing crawling modes by recruiting the four inter-mode shared synergy structures related to the swing function of each limb in different orders, and realize the two-limb swing crawling modes by recruiting synchronously two intra-mode shared synergy structures. The research results of the muscle synergy analysis on the eight specific inter-limb coordination modes, on the one hand, provide a basis for muscle synergy hypothesis from the perspective of crawling motion, on the other hand, also provide a possible explanation for the choice of the inter-limb coordination mode in human crawling.

## 1. Introduction

Over the past decades, the question how the central nervous system (CNS) controls muscles to perform specific motor tasks has attracted widespread attention (d’Avella et al., 2003, 2006, 2011; Tresch and Jarc, 2009; Tang et al., 2015; Taborri et al., 2018; Xiong et al., 2018). Lots of researches show that vertebrates generate motor behaviors by a combination of rudimentary motor modules (Tresch et al., 2002; Flash and Hochner, 2005; Bizzi et al., 2008; Cheung et al., 2012). Muscle synergy, as one kind of the motor modules, has been used for many years to explain how the CNS achieves the neuromuscular control of different movements (d’Avella et al., 2003, 2006, 2011; Tresch and Jarc, 2009; Tang et al., 2015, 2017; Taborri et al., 2018; Xiong et al., 2018). Muscle synergy hypothesis states that the CNS generates motor commands by recruiting a group of muscles to work together instead of a single muscle (Saltiel et al., 2001; d’Avella et al., 2003; Bizzi et al., 2008; Tang et al., 2017), that is, a group of muscles are continuously activated in space and time (Torres-Oviedo et al., 2006; Taborri et al., 2018). Although this theory has been confirmed by many studies, there are also opposing voices (Kurtzer et al., 2006; Kutch et al., 2008; Tresch and Jarc, 2009; Valero-Cuevas et al., 2009), and more evidence is needed.

As an electrophysiological signal related to muscle contraction, surface electromyography (sEMG) signal has unique advantages in motion analysis, as it carries abundant information related to muscle activation and neuromuscular control mechanism (Tang et al., 2017; Gao et al., 2018). Muscle synergy can be extracted from sEMG data recorded from relative muscles by using blind source separation algorithms such as non-negative matrix factorization (NNMF) (Lee and Seung, 1999; Ting and Macpherson, 2005), principal component analysis (PCA) (Ting and Chvatal, 2010), independent components analysis (ICA) (Hyvärinen and Oja, 2000), and factor analysis (FA) (Kieliba et al., 2018; Rabbi et al., 2020) etc. According to Rabbi et al. (2020), NNMF is the most appropriate algorithm for muscle synergy extraction. When NNMF is applied to a sEMG data matrix, two matrices named muscle synergy structure matrix and recruitment curve matrix, respectively, can be obtained (Cheung et al., 2012; Wojtara et al., 2014; Taborri et al., 2018). The synergy structure matrix consists of weight vectors representing each muscle’s contribution to a particular synergy, while the recruitment curve matrix consists of vectors representing the recruited pattern of a particular synergy (Wojtara et al., 2014; Taborri et al., 2018).

As the earliest quadruped movement mastered by most humans, crawling is closely related to various functions of the human body, including spatial concepts, eye-hand coordination, vestibular processing, balance and equilibrium, spatial awareness, tactile input, kinesthetic awareness, and social maturation (McEwan et al., 1991; Kretch et al., 2014). Crawling can be used as an evaluation object for clinicians to judge patients’ motor ability subjectively or a beneficial item for patients to carry out motor function rehabilitation training (Xiong et al., 2021). For both clinical diagnosis and rehabilitation training of motor dysfunction, it is of great significance to understand the inter-limb coordination mechanisms of human crawling movement.

In fact, the muscle synergy analysis based on sEMG signals has been applied to the analysis of human crawling in related researches (Chen et al., 2017; Gao et al., 2018; Xiong et al., 2018; Li et al., 2019). Chen et al. conducted a study to explore the intra-limb coordination mechanism of adult crawling (Chen et al., 2017). They found that two synergies could be extracted from each limb to represent the swing and stance phases during crawling, the intra-limb muscle synergy was relatively stable, and the crawling speed affected the synergy structures slightly but changed the recruitment levels significantly. Xiong et al. explored the inter-limb muscle synergies of children crawling, and found that infants with confirmed developmental delays had fewer muscle synergies (Xiong et al., 2018). Gao et al. used synergistic recruitment of sEMG frequency components to represent the myoelectric activity, three patterns (corresponding to low-frequency range, medium-frequency range and high-frequency range respectively) of sEMG oscillation synergies in each muscle of both typically developed infants and infants with cerebral palsy were extracted when they crawled on hands-and-knees (Gao et al., 2018). Taking hands-and-knees crawling of healthy children and children with cerebral palsy as research object, Li et al. explored the abilities of synchronous and time-varying muscle synergy analysis methods in distinguishing normal and abnormal crawling movements (Li et al., 2019).

All the researches mentioned above show that the muscle synergy hypothesis has been verified in human crawling, and that muscle synergy analysis can be used to reveal the abnormal neuromuscular control strategies in crawling movement of patients with dyskinesia. In existing researches the inter-limb coordination modes of crawling are mainly divided into three categories, namely: (1) pace-like mode, in which the ipsilateral limbs move together; (2) trot-like mode, in which the diagonal limbs move together; (3) no-limb-pairing mode, in which all limbs move at regular intervals (Patrick et al., 2009). Since trot-like mode and pace-like mode are the most preferred crawling modes, most of the researches has focused mainly on these two modes (Freedland and Bertenthal, 1994; Patrick et al., 2012; Righetti et al., 2015; Chen et al., 2017; Ma et al., 2017; Cole et al., 2019). In the meanwhile, the muscle synergy analysis on human crawling is mainly based on participant’s self-selected inter-limb coordination mode, namely, the trot-like mode and pace-like mode (Chen et al., 2017; Gao et al., 2018; Xiong et al., 2018; Li et al., 2019). However, crawling is a complex four-beat gait. Theoretically, humans can complete crawling with a variety of inter-limb coordination modes. That is to say, the previous muscle synergy analyses on crawling movement can only partially reflect the neuromuscular control mechanism of the CNS due to the limited number of inter-limb coordination modes involved.

Different from the relevant studies, which focus on muscle synergy analysis of autonomously selected coordination modes between limbs, the novelty of the crawling research carried out in this paper is that it expands the research object from the three typical modes to eight modes, so as to realize the goal of revealing in-depth the neuromuscular control mechanism of human crawling. In particular, taking hands-knees crawling as the research object, eight specific inter-limb coordination modes are defined according to the swing sequence of limbs. The NNMF algorithm is adopted to extract muscle synergies from sEMG data firstly. Then the intra-mode shared synergies among participants and the inter-mode shared synergies among the eight inter-limb coordination modes are extracted by using the hierarchical clustering method. Specifically, the shared synergy structures and shared recruitment curves are extracted separately. Finally, based on the definition of the eight inter-limb coordination modes and the characteristics of intra-mode/inter-mode shared synergy structures and recruitment curves, the control mechanism of CNS on human crawling are deeply analyzed.

## 2. Materials and methodology

**Figure 1:**
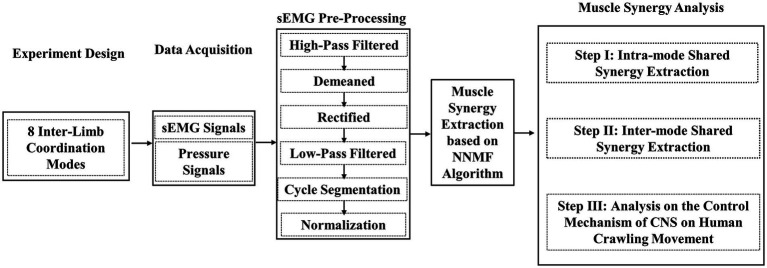
Flowchart of the research on muscle synergy analysis of eight inter-limb coordination modes during human hands-knees crawling movement.

Figure 1 gives the research route of this paper, which mainly includes the following steps: 1) the definition of eight specific inter-limb coordination modes; 2) crawling sEMG data collection and pre-processing; 3) muscle synergy extraction based on NNMF algorithm; and 4) exploration on the control mechanism of CNS on human crawling through muscle synergy analysis. The specific implementations are introduced in detail below.

### 2.1. Definition of eight inter-limb coordination modes

This study mainly focuses on the inter-limb coordination modes that two limbs swing together or four limbs swing one by one at the same intervals. When two limbs swing together, there are two possible modes: 1) right hand and right knee swing together, then left hand and left knee swing together, namely “right hand, right knee → left hand, left knee”; and 2) right hand and left knee swing together, then left hand and right knee swing together, namely “right hand, left knee→ left hand, right knee.” According to Patrick’s study (Patrick et al., 2009), these two crawling modes correspond to pace-like mode and trot-like mode, respectively. When limbs swing one by one, there are 6 types of inter-limb coordination modes. Therefore, as shown in Table 1, the eight specific inter-limb coordination modes (M1–M8) are defined according to the swing sequence of limbs.

**Table 1:** Eight inter-limb coordination modes during crawling.

Modes	Swing sequence of limbs
M1	Right hand, right knee → left hand, left knee
M2	Right hand, left knee → left hand, right knee
M3	Right hand→ left knee→ right knee → left hand
M4	Right hand→ right knee→ left knee → left hand
M5	Left knee→ right hand→ right knee → left hand
M6	Left knee→ right knee→ right hand→ left hand
M7	Right knee→ right hand→ left knee → left hand
M8	Right knee→ left knee→ right hand → left hand

### 2.2. Crawling data acquisition

Ten healthy adult participants (P1 to P10, 7 males, 3 females, 23.90 ± 0.88 years) participate in the crawling data collection experiment. All participants have no history of neuromuscular diseases or motor dysfunctions, are informed of the study content, and sign an informed consent form. The Ethics Review Committee of Anhui Medical University approves this study (No. PJ 2014-08-04).

**Figure 2:**
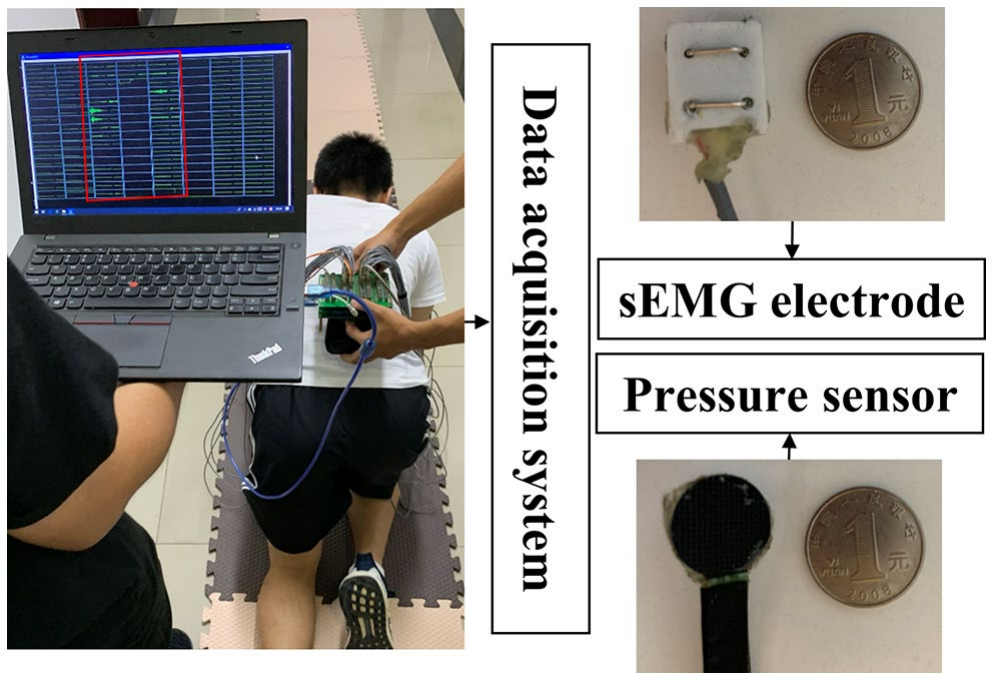
Illustration of the home-made data acquisition system.

**Figure 3:**
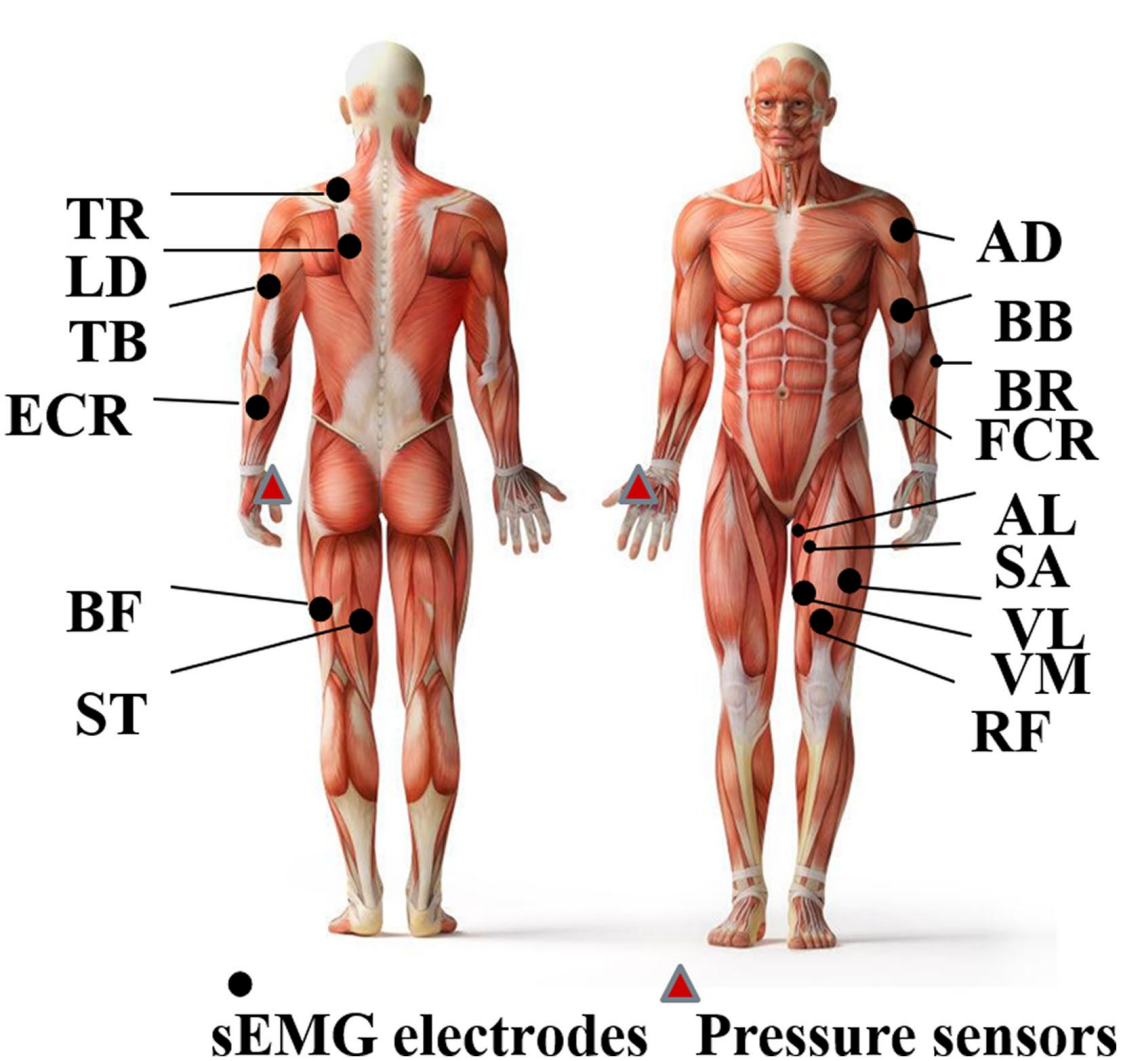
Placement of sEMG sensors and pressure sensors.

A laboratory-made data acquisition system (ADS 1626, Texas Instruments, band pass filter: 20–500 Hz; A/D resolution: 18-bit; gain: 1,100 times), as shown in Figure 2, is used to collect the sEMG data frm 30 pieces of limb and trunk muscles related to crawling movement and pressure signals from the left and right palms simultaneously. The active sEMG electrodes are 23 mm length and 20 mm width, embedded with bipolar separating silver wires (the interval is 10 mm). The thin-film piezoresistive pressure sensors RP-C18.3-ST (18.3 mm diameter) produced by the company WAAAX) is adopted to collect pressure signals. As shown in Figure 3, the target muscles include the trapezius (TR), anterior deltoid (AD), latissimus dorsi (LD), biceps brachii (BB), triceps brachii (TB), brachioradialis (BR), extensor carpi radialis (ECR), flexor carpi radialis (FCR), rectus femoris (RF), vastus lateralis (VL), vastus medialis (VM), sartorius (SA), adductor longus (AL), biceps femoris (BF), and semitendinosus (SE). The electrode placement is performed according to SENIAM guidelines (Hermens et al., 2000). Since the most important function of the pressure signal is to divide the crawling cycle, the pressure sensors are placed only on the flexor pollicis brevis of left palm and right palm, respectively, and are fixed by the kinesiology tape. Both the sEMG electrodes and pressure sensors are connected to the data collection system via a USB. To avoid power frequency interference, a lithium battery is used to power the system. The signal sampling rate of each channel is set to 1,000 Hz. To ensure good contact with the skin, the electrodes are fixed by the kinesiology tape and double-sided tape (hollowed out by a punch).

To ensure that participants could complete crawling movement at the same speed, an audio guidance file is produced for each inter-limb coordination mode to guide the limb swinging order, with a rhythm of 2.33 s/cycle. Each audio guidance file contained at least 20 cycles. During the data collection experiment, the participants crawl on a foam pad (11.2 meters long and 0.8 meters wide). A mobile phone broadcast the audio guidance file, and participants complete crawling movements according to the recording prompts. Before the data collection, participants learn the rules (the waist should be parallel to the ground, the eyes should look straight ahead, and the limbs should swing naturally) for executing the crawling movement in the eight inter-limb coordination modes, and practice until they are proficient (can crawl according to the rhythm given by the recording file for more than 10 cycles without making mistakes).

During the experiment, at least two operators are needed, one operator moves with the electronics and the other moves with the computer since data transfer is carried out by USB. Artifacts may be introduced during crawling movement. In order to reduce the impact of artifacts, the following efforts have been made: (1) Before the data acquisition experiments, operators will make sure that the equipment wiring is firm and stable. On the one hand, the operators will carefully check each connection interface, especially the interface of the acquisition system and the interface of the computer, which connected by USB cable, to ensure that the connection is stable; on the other hand, the data transmission lines of sEMG electrodes and pressure sensors may interfere with each other, which may cause artifacts. In order to solve this problem, the data transmission lines of the sEMG electrodes and the pressure sensor of the same limb will be fixed with adhesive tape as shown in Figure 2; (2) During the data acquisition experiments, the experimental data of a crawling mode completed by each participant will be stored independently. As shown in Figure 2, one operator will observe the real-time signal on the computer. Once the obvious artifact noise is observed, the experimental data of this crawling mode will be discarded. And the data collection experiment will not be restarted until the artifact noise is eliminated; (3) In the data preprocessing stage, the sEMG signal will be high-pass filtered, demeaned, and low-pass filtered to obtain the envelope. These preprocessing steps of sEMG signals can further reduce the influence of artifact noise.

In each trial, participants complete the crawling task in order from M1 to M8. For each inter-limb coordination mode, the participants should complete at least 10 continuous crawling cycles without any action errors. The participants are asked to rest for about 5 min after a 10-min crawling movement to avoid muscle fatigue. Since participants are more likely to make action errors at the beginning or end of a crawling movement, the first and last two crawling cycles are abandoned. Consequently, for each inter-limb coordination mode, there are at least 8 effective crawling cycles for each participant. Table 2 shows the total number of crawling cycles across all the 8 modes (M1–M8) for each participant. All the data are saved in a laptop and are analyzed offline in MATLAB 2019a.

**Table 2:** The number of crawling cycles of each participant at three speeds.

	P1	P2	P3	P4	P5	P6	P7	P8	P9	P10
M1	12	10	12	10	9	10	12	11	10	11
M2	14	9	10	10	10	11	10	10	10	11
M3	13	12	8	12	9	12	12	12	13	14
M4	14	11	9	10	10	11	13	11	12	17
M5	13	8	12	14	13	14	13	12	11	14
M6	14	9	11	13	11	13	10	12	11	15
M7	12	9	17	14	10	11	15	11	12	13
M8	13	9	11	13	13	12	13	11	12	14

### 2.3. Data preprocessing

**Figure 4:**
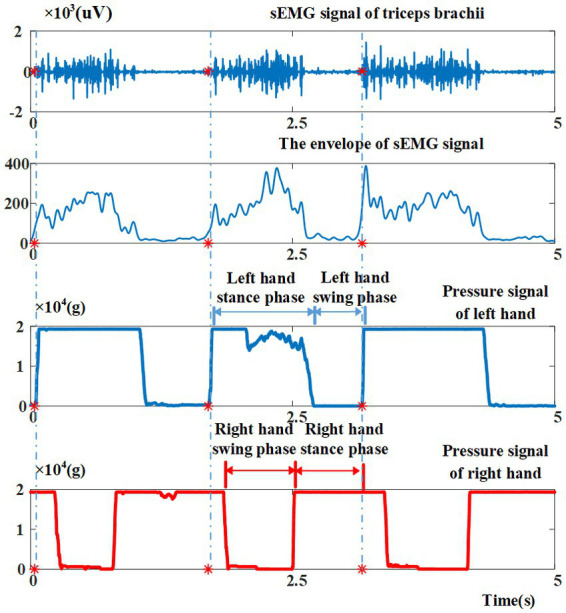
Illustrations of sEMG signal and pressure signals collected during crawling movement, as well as the representative phases, namely left hand stance phase, left hand swing phase of crawling cycle, right hand swing phase, right hand stance phase. Red asterisks indicate the starting points of crawling cycles.

Figure 4 shows a channel of sEMG signal and two channels of pressure signal collected during a crawling movement. The sEMG signals are high-pass filtered (window-based finite impulse response filter, 100th order, cutoff 30 Hz) to remove low-frequency noise signals, and then are demeaned, rectified, and low-pass filtered (window-based finite impulse response filter, 100th order, cutoff 10 Hz) to extract the envelope. Immediately after, the crawling cycles are segmented from the continuous signals. In this study, the time when the left hand ends its swing phase is considered to be the beginning of a crawling cycle. As shown in Figure 4, at the moment the left palm touching the foam pad (corresponding to the start of the left hand stance phase), the value of the pressure signal jumps from minimum to maximum in a short time, and drops to a minimum at approximately 2/3 cycles. Therefore, the pressure signal of the left palm is used for the segmentation of the crawling cycles. More specifically, a sliding window (length: 100 ms, increment: 1 ms) is adopted to find the crawling cycle starting points according to the following criterion: the first derivative on the left of the starting point is zero and the first derivative on the right of the starting point is greater than zero. When one crawling cycle starting point is found, the sliding epoch window is shifted 500 points (500 ms) to find the next crawling cycle starting point. Finally, the length of the crawling cycle is down-sampled to 1,000 points (1 s) and the sEMG amplitude per muscle is normalized to the activation peak across all crawling cycles in a given inter-limb coordination mode (Clark et al., 2010; Tang et al., 2015).

### 2.4. Muscle synergy extraction based on non-negative matrix factorization algorithm

In order to verify muscle synergy theory, that is, to explore whether the CNS controls crawling by recruiting muscle groups or individual muscles, this study carries out muscle synergy extraction on the crawling data of the 10 participants in all the eight crawling modes firstly. To extract the muscle synergy, the NNMF algorithm is applied to the preprocessed sEMG signal of each crawling cycle (Lee and Seung, 1999). As shown in Equation (1), a sEMG matrix *M* (*m* × *n*, *m*: the number of muscles, *n*: the length of the crawling cycle, *m* = 30, *n* = 1,000 in this study) can be decomposed into a muscle synergy structure matrix *W* (*m* × *s*) and the corresponding recruitment curve matrix *C* (*s* × *n*). Where *E* is the error matrix between *M* and the product of *W* and *C* and *s* is the number of muscle synergies. Each column of *W* represents one muscle synergy, and each row of *C* represents the recruitment curve of the corresponding muscle synergy. According to the NNMF theory (Lee and Seung, 1999), *W* and *C* start with a random non-negative initialization, and then are iterated by gradient descent until *E* drops to an expected value. The minimum number of muscle synergies is determined by the variability accounted for (VAF) shown in Equation (2). In this study, the threshold is set to 0.9.


(1) $$M^{m\times n}=W^{m\times s}C^{s\times n}+E$$

(2) $$VAF=1-\frac{\left\|E^{2}\right\|}{\left\|M^{2}\right\|}$$

Pearson correlation coefficients, which can be calculated according to Equations (3) and (4), are adopted to evaluate the similarity of the two muscle synergies from the perspectives of structures and recruitment curves.


(3) $$r_{W∼W\prime }=\frac{m\sum_{i=1}^{m}W_{i}W\prime_{i}-\sum_{i=1}^{m}W_{i}\sum_{i=1}^{m}W\prime_{i}}{\sqrt{m\sum_{i=1}^{m}W_{i}{\;}^{2}-{\left(\sum_{i=1}^{m}W_{i}\right)}^{2}}\cdot \sqrt{m\sum_{i=1}^{m}W\prime_{i}{\;}^{2}-{\left(\sum_{i=1}^{m}W\prime_{i}\right)}^{2}}}$$

(4) $$r_{C∼C\prime }=\frac{n\sum_{i=1}^{n}C_{i}C\prime_{i}-\sum_{i=1}^{n}C_{i}\sum_{i=1}^{n}C\prime_{i}}{\sqrt{n\sum_{i=1}^{n}C_{i}{\;}^{2}-{\left(\sum_{i=1}^{n}C_{i}\right)}^{2}}\cdot \sqrt{n\sum_{i=1}^{n}C\prime_{i}{\;}^{2}-{\left(\sum_{i=1}^{n}C\prime_{i}\right)}^{2}}}$$

In this study, we make the assumption that one participant would use the same neuromuscular control mechanism to complete multiple consecutive crawling cycles in the same crawling mode, and consider the cycle-to-cycle variability to be noise. To remove this noise from the analysis, the following process are adopted to extract muscle synergy from one type of crawling modes with L crawling cycles:

Step 1: Set s = 0;
Step 2: s = s + 1, extract muscle synergy and calculate the VAF value according to Equation (2) for each cycle, and compute the mean VAF by averaging the values of all L cycles;
Step 3: Check if the mean VAF is higher than 0.9. If yes, go to Step 4; else, go to Step 2;
Step 4: Stop the iteration and determine *s* as the number of muscle synergies;
Step 5: Align synergy matrices between cycles and average the *L* aligned synergy matrices as the final extracted muscle synergies of this crawling mode.

Aligning synergy matrices of different cycles is realized as follow. For the synergy structures matrices A = [$W_{a1},W_{a2},\ldots ,W_{as}$] and B = [$W_{b1},W_{b2,}\ldots ,W_{bs}$] extracted from two different cycles respectively, take matrix A as reference, reorder the synergies in matrix B, and calculate the total similarity of A and B as $r_{total}=\sum_{i=1}^{s}r_{W_{ai}\sim W_{bi}}$. Among the all reordered matrices of B, taking the matrix $B^{\prime }\;$ which get the maximum total similarity as matching matrix of A, namely the aligned synergy structure matrix. Accordingly, the corresponding recruitment curve matrix of $B^{\prime }\;$ is considered as the aligned recruitment curve matrix.

### 2.5. Muscle synergy analysis scheme based on hierarchical clustering method

In order to in-depth reveal the control mechanism of the CNS in crawling, the following three questions are intended to be answered by muscle synergy analysis, namely: (1) Are there shared synergies when different participants crawl with the same inter-limb coordination mode? (2) Are there shared synergies between different inter-limb coordination modes? (3) Do these shared synergies reflect neuromuscular control mechanisms of the CNS during crawling movement? For these purposes, the three-step muscle synergy analysis scheme based on the hierarchical clustering method is performed.

#### 2.5.1. Hierarchical clustering method

Hierarchical clustering is a greedy algorithm, which is usually implemented by agglomerative or divisive strategies (Murtagh and Contreras, 2017). In this study, the agglomerative strategy whose clustering process is equivalent to building a binary tree (dendrogram, in which the observations are all leaf nodes) is adopted. Specifically, for an observation matrix *X =* [*X*_1_, *X*_2_, …, *X_N_*]^T^, following operations are carried out to complete the hierarchical clustering on the *N* observation vectors:

Step 1: Initialize *k* = 2. Calculate the Euclidean distances between each two observations in observation matrix *X* according to Equation (5);
Step 2: Remove from *X* the two observations and with the smallest Euclidean distance and construct a new matrix *Y* = [*Y*_1_ = *X*_i_,*Y*_2_ = *X*_j_];
Step 3: Set *k* = *k* + 1 and calculate the Euclidean distances between *Y* and the remaining *N*-*k* + 1 observations in *X* according to Equation (6);
Step 4: Remove from *X* the observation $\;X_{c}\;\left(1\leq c\leq N\right)$ which has the smallest Euclidean distance from *Y* and expand *Y* = [*Y*_1_,*Y*_2_.., *Y*_k_ =  *X*_c_]
(5) $$d\left(X_{i},X_{j}\right)=\sqrt{\sum_{a=1}^{n}{\left(X_{ia}-X_{ja}\right)}^{2}}\left(1\leq i\leq N,1\leq j\leq N\right)$$
  d(*Y*, *X*_c_) = min {d(*Y*_1_, *X*_c_), d(*Y*_2_, *X*_c_),…, d(*Y*_k_, *X*_c_)}   (6)
Step 5: Repeat Step 3 to Step 4 until all observations in *X* are removed.

**Figure 5:**
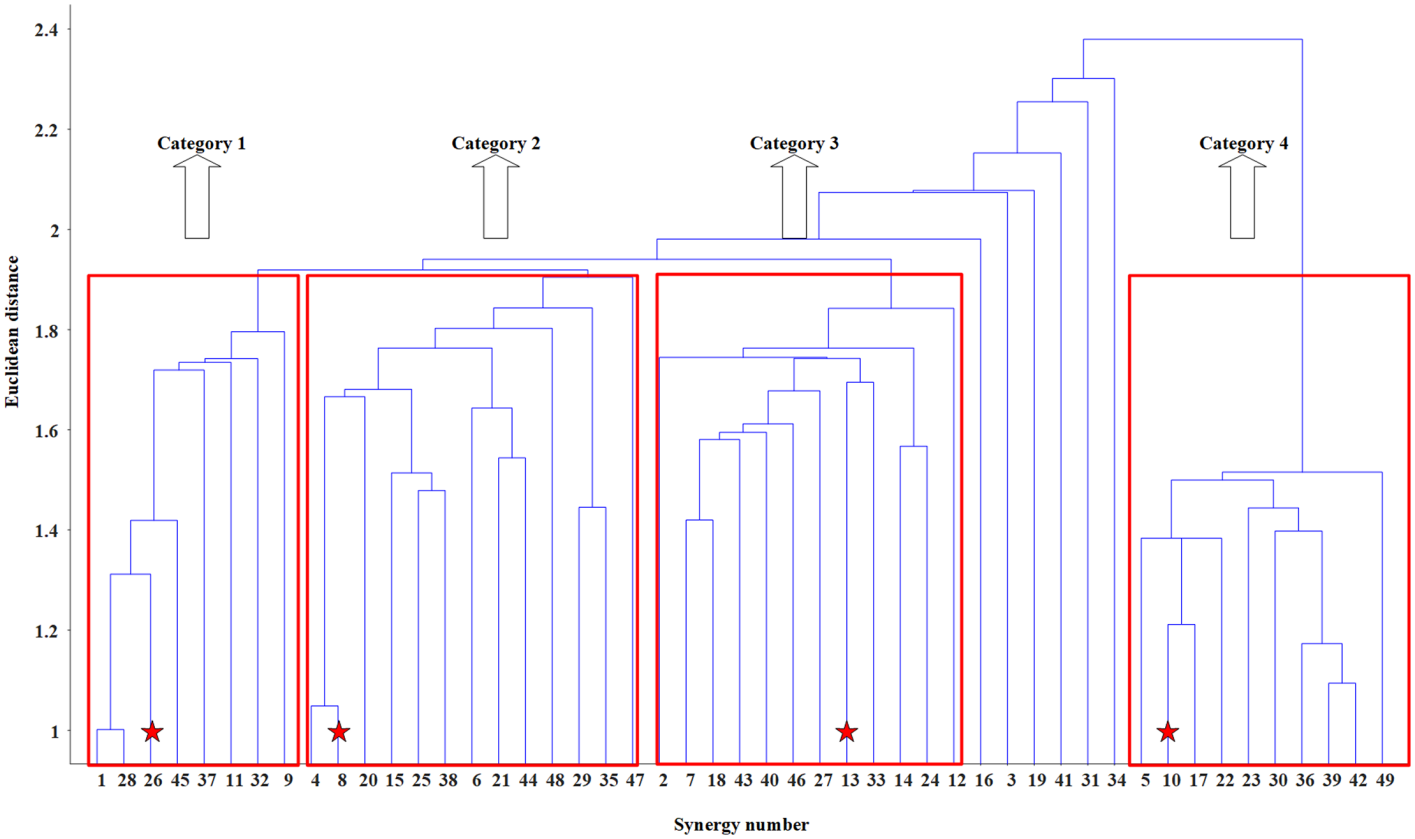
The dendrogram obtained by the hierarchical clustering method in M3. The centroids of the four categories are marked by red five-pointed stars.

Finally, *Y* matrix with elements sorted by Euclidean distance is obtained. The single linkage method, whose principle is to cluster the two observations (or clusters) with the smallest Euclidean distance into one cluster each time, can be applied on *Y* to obtain the dendrogram as shown in Figure 5.

#### 2.5.2. Intra-mode shared synergies extraction

To answer the question of whether there exist shared synergies when different participants crawl with the same inter-limb coordination mode, the intra-mode shared synergy structures and recruitment curves of 10 participants in each crawling mode are extracted in this study.

For the inter-limb coordination mode *M_i_* (*i* = 1, 2, …, 8), assume that the extracted synergy number of participant *Pj* (*j* = 1, 2, …,10) is *N_ji_* and the total synergy number of the 10 participants is *T_i_*=$\sum_{j=1}^{10}N_{ji}$, synergies extracted from participants P1 ~ P10 are sequentially numbered 1 to *T_i_*. The synergy structure vectors among participants in each inter-limb coordination are combined into a T_i_*30 structure matrix *W_Ai_*, and the synergy recruitment curves are combined into a *T_i_* *1,000 recruitment curve matrix *C_Ai_*. The hierarchical clustering method adopting agglomerative strategy mentioned above is applied on *W_Ai_* and *C_Ai_, respectively.*

After the dendrograms are obtained, the synergy structures or recruitment curves are classified into several categories based on an appropriate Euclidean distance. And the appropriate Euclidean distance needs to meet the following rules: (1) the number of synergies classified into the same category is larger than a threshold TH_1; (2) the number of categories is larger than a threshold TH_2. For each category, the centroid needs to be determined. Taking 0.6 as the similarity threshold (Ranganathan et al., 2016), the criterion of being the centroid is that the synergy has the greatest similarity with the remaining synergies, that is, the quantity greater than 0.6 is the largest in this category. After determining the centroids of all categories, synergies with a similarity less than 0.6 to the centroids are discarded. At last, the average of at least four synergies with similarity greater than 0.6 is used to represent this category of muscle synergy, that is, the intra-mode shared synergy.

#### 2.5.3. Inter-mode shared synergies extraction

To answer the question of whether there exist shared synergies between different crawling modes, the inter-mode shared synergy structures and recruitment curves of the eight modes are extracted from the intra-mode shared synergies. In particular, the intra-mode shared synergy structures (or recruitment curves) extracted in the previous step are combined into a l*30 (or l*1,000) matrix, where *l* is the number of the intra-mode shared synergy structures (or recruitment curves). The extraction process of inter-mode shared synergies is similar with that of the intra-mode shared synergies. The average of at least four intra-mode shared synergy structures (or recruitment curves) with similarity greater than 0.6 is used to express this category of muscle synergy, that is, the inter-mode shared synergy.

#### 2.5.4. Muscle synergy analysis scheme for exploring the neuromuscular control mechanism of the CNS

In order to explore the neuromuscular control mechanism of the CNS in human crawling, the characteristics of intra-mode/inter-mode shared synergy structures and recruitment curves need to be analyzed from the following aspects: On the one hand, to figure out whether more structural features or temporal features are shared among different participants when they crawl with the same inter-limb coordination mode, the proportions of the intra-mode shared synergy structures and recruitment curves among the extracted muscle synergies of the 10 participants is calculated and analyzed; On the other hand, in order to reveal the possible reasons why humans choose different inter-limb coordination modes during crawling, the functions achieved by inter-mode/intra-mode shared synergies, and their recruitment pattern in single-limb swing crawling modes M3 ~ M8 and two-limb swing crawling mode M1 and M2 will be analyzed in-depth.

## 3. Results and analysis

### 3.1. The number of muscle synergies extracted during crawling movement

Table 3 gives the number of muscle synergies extracted from 10 participants in each inter-limb coordination mode. It is easy to observe that, 4 to 7 synergies are extracted from each participant in each inter-limb coordination mode. When a participant completed a crawling task with a specific inter-limb coordination mode as one crawling trial, there are a total of 80 crawling trials in this study. Of these, 40% recruits 5 synergies, 30% recruits 6 synergies, 28.75% recruits 6 synergies, and only 1.25% recruits 7 synergies. These results support the muscle synergy hypothesis to some extent, namely, CNS controls the inter-limb coordination modes during crawling movement by recruiting a certain amount of muscle synergies, rather than a single muscle.

**Table 3:** The number of extracted muscle synergies, the intra-mode shared synergy structures and recruitment curves (*SUM: the total number of the muscle synergies extracted from each crawling mode; IAS: the total number of intra-mode shared synergy structures; IAR: the total number of intra-mode shared recruitment curves).

	P1	P2	P3	P4	P5	P6	P7	P8	P9	P10	SUM	IAS	IAR
M1	5	6	5	6	4	7	4	4	6	5	52	19	37
M2	5	6	5	6	4	6	4	5	5	5	51	21	39
M3	6	6	5	5	4	6	4	4	5	4	49	33	41
M4	6	6	4	6	4	5	4	4	5	4	48	33	44
M5	6	6	5	6	5	6	4	4	6	5	53	35	47
M6	6	6	4	6	4	5	5	4	5	5	50	34	47
M7	6	5	4	6	4	5	5	5	5	5	50	28	42
M8	6	5	4	6	5	5	4	5	5	5	50	30	46

### 3.2. Demonstration of synergy clustering based on the hierarchical clustering method

To give readers a clear understanding of the specific process of synergy clustering based on the hierarchical clustering method, this section takes synergy structure clustering of the inter-limb coordination mode M3 among participants as an example to explain in detail. As shown in Table 3, a total of 49 synergies are extracted from all 10 participants in mode M3. Of those, 6 synergies extracted from P1 are numbered from 1 to 6, 6 synergies extracted from P2 are numbered from 7 to 12, and so on, 4 synergies extracted from P10 are numbered from 46 to 49. The synergy structure vectors among participants are combined into a 49*30 structure matrix *W_A3_*, and the hierarchical clustering method adopting agglomerative strategy is applied to it.

**Figure 6:**
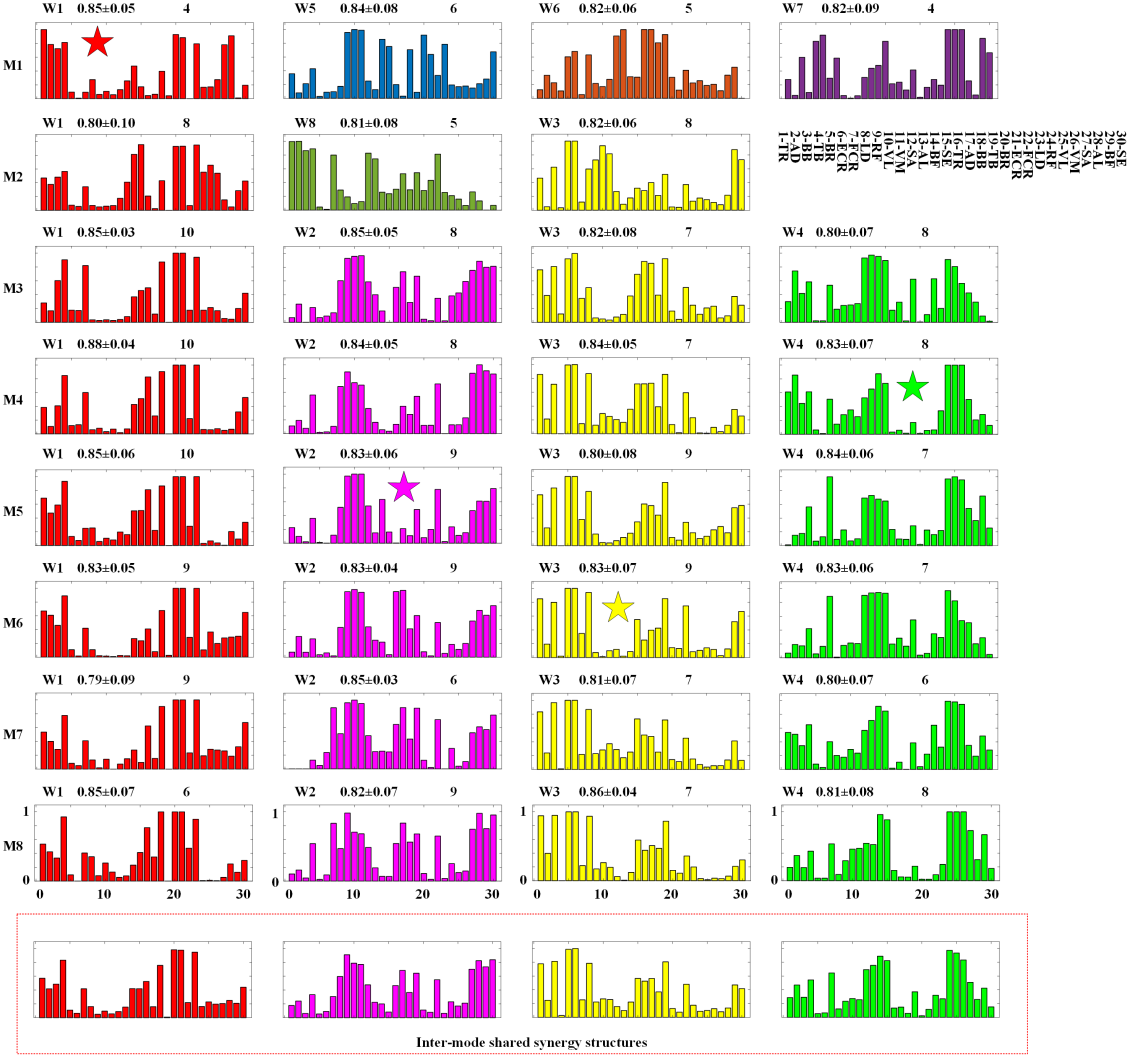
Intra-mode synergy structures and inter-mode synergy structures. M1 ~ M8: Inter-limb coordination modes. W1 ~ W8: synergy structures.

The dendrogram is obtained after using hierarchical clustering method. The appropriate Euclidean distance threshold is set to 1.90 to distinguish different categories, since it meets the rules: 1) the number of synergies classified into the same category is above 8, namely TH_1 = 8; 2) the number of categories is above 2, namely TH_2 = 2. As shown in Figures 5, 6 of the 49 synergy structures are discarded, and the remaining 43 synergy structures are divided into four categories. When using the criterion mentioned in MATERIALS AND METHOD to determine the centroid of each category, the centroid of Category 1 is confirmed to be the 26th synergy structure, the centroid of Category 2 is confirmed to be the 8th synergy structure, the centroid of Category 3 is confirmed to be the 13th synergy structure, and the centroid of Category 4 is the 10th synergy structure. After the centroids are determined, synergy structures with similarity less than 0.6 to the centroids is discarded, and those with similarity higher than 0.6 are averaged as the intra-mode shared synergy structure. The results of the synergy structure clustering in M3 are shown in the third row of Figure 6.

### 3.3. Extraction results of intra-mode shared synergies and inter-mode shared synergies

#### 3.3.1. Intra-mode shared synergies

**Figure 7:**
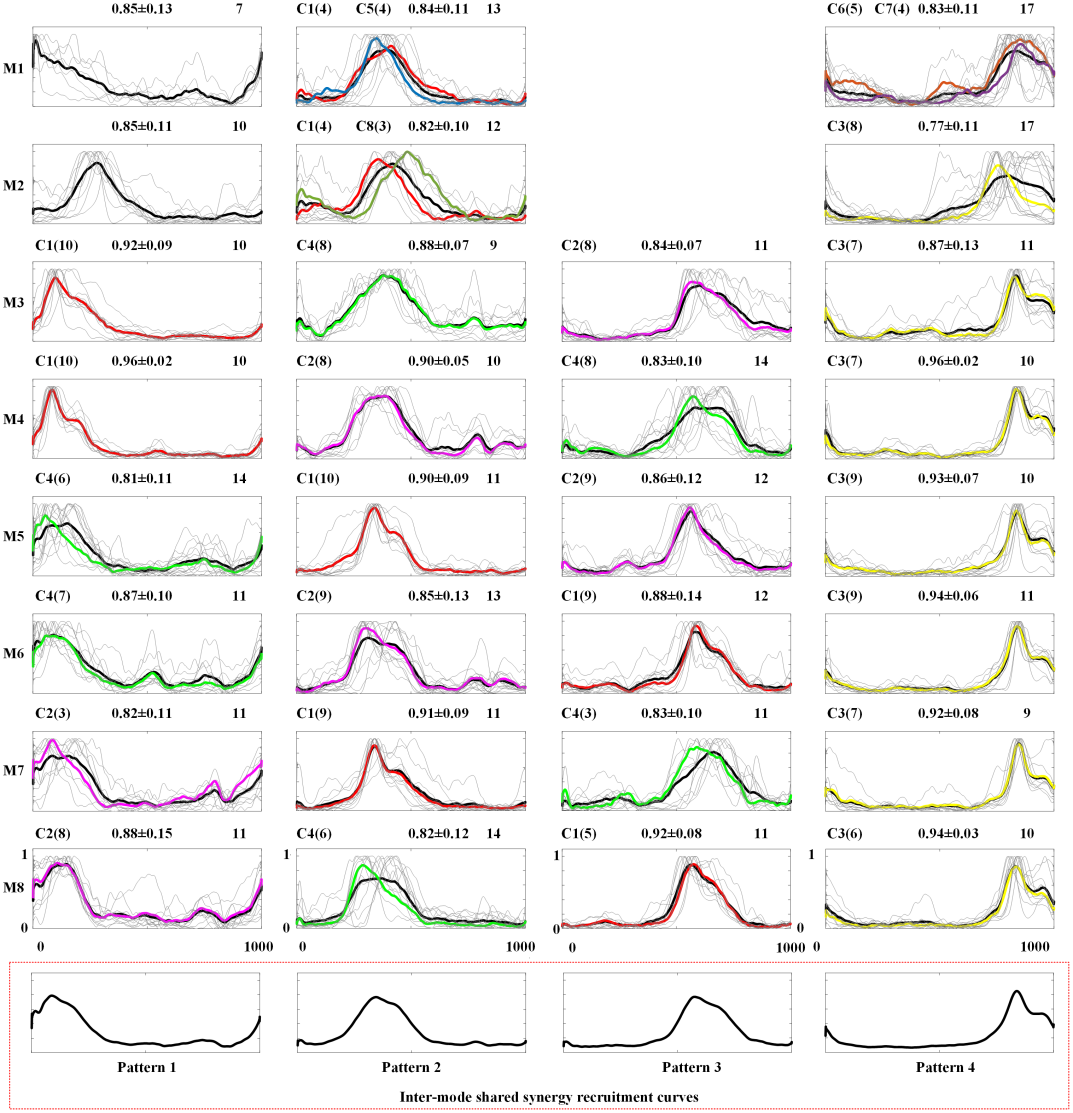
Intra-mode recruitment curves and inter-mode recruitment curves. C1 ~ C8: recruitment curves corresponding to W1 ~ W8.

In the clustering analysis experiment carried out to extract intra-mode shared synergies, for each inter-limb coordination mode, the muscles synergies extracted from all the 10 participants are clustered using the hierarchical clustering method, Figures 6, 7 show the clustering results of synergy structures and recruitment curves, respectively.

In Figure 6, the first eight rows give the intra-mode shared synergy structures for each mode, and the last row shows the inter-mode shared synergy structures. In each subgraph, the horizontal axis corresponds to the 30 muscles listed in the upper-right of the figure (1 ~ 15 correspond to muscles of the left body and 16 ~ 30 correspond to muscles of the right body). The number in the upper right represents the number of muscle synergies classified into this type of intra-mode shared synergy structure. In Table 3, the total number of the synergy structures identified as intra-mode shared synergy structures in each crawling mode is given as IAS. The values in the middle are the mean and standard deviation of the similarities between these synergy structures and the averaged synergy structure. It can be found that 3 ~ 4 intra-mode shared synergy structures are extracted from each inter-limb coordination mode, specifically, M1, M3, M4, M5, M6, M7, and M8 have 4, and M2 has 3. Therefore, there are 31 intra-mode shared synergy structures among the eight crawling modes in total.

In Figure 7, the first eight rows give the intra-mode shared recruitment curves for each mode, and the last row shows the inter-mode shared synergy recruitment curves. In each subgraph, the number in the upper-right represents the number of recruitment curves classified into this type of intra-mode shared recruitment curves. In Table 3, the total number of the recruitment curves identified as intra-mode shared recruitment curves in each crawling mode is given as IAR. The mean and standard deviation of the similarities between these synergy recruitment curves and the averaged recruitment curve are also given. The thin gray lines depict these similar recruitment curves, and the thick black line depicts the averaged recruitment curve. It can be found that 3 ~ 4 intra-mode shared synergy recruitment curves have been extracted from each inter-limb coordination mode, specifically, both M1 and M2 have 3, M3 ~ M8 all have 4. Therefore, there are 30 intra-mode shared recruitment curves among the eight crawling modes in total.

#### 3.3.2. Inter-mode shared synergies

In the clustering analysis experiment for extracting shared synergies between the 8 inter-limb coordination modes, the 31 intra-mode shared synergy structures are further clustered, and 4 inter-mode shared synergy structures and 4 specific synergy structures are obtained. In Figure 6, the 4 inter-mode shared synergy structure are named W1 ~ W4 and depicted in red, pink, yellow and green colors, respectively. That is, subgraphs with the same color represent the same inter-mode synergy structure. The rest 4 synergy structures are named W5 ~ W8. In particular, W1 in M1, W2 in M5, W3 in M6, and W4 in M4 are the centroids of the four categories, which are marked with a five-pointed star.

When the 30 intra-mode shared recruitment curves are further clustered, four inter-mode shared recruitment curves, which also are defined as Pattern 1 to Pattern 4, are obtained. As shown in Figure 7, Pattern 1 mainly represents the synergy recruitment in the first quarter of the crawling cycle. Accordingly, Pattern 2, Pattern 3 and Pattern 4 represent the synergy recruitment of the second quarter, the third quarter, and the last quarter of crawling cycle, respectively.

In order to facilitate following exploration on the neuromuscular control mechanisms of human crawling movement, in each subgraph of Figure 7, the number of the synergy recruitment curves C1 ~ C8 corresponding to synergy structures W1 ~ W8 are also given, and the averaged recruitment curve of *C_i_* is depicted in the same color with *W_i_* (*i* = 1, 2, …, 8).

### 3.4. Analysis on the neuromuscular control properties of CNS during human crawling

Combined with the definitions of the eight inter-limb coordination modes and the shared synergy extraction results shown in Figures 6, 7 and Table 3, the following neuromuscular control properties of human crawling can be summarized:

At first, through the analysis of the intra-mode shared synergies, it can be found that the control of CNS on human crawling has better commonality in the aspect of synergy recruitment pattern than synergy structure. That is to say, when different participants complete the same crawling mode, they will share more temporal features. As shown in Table 3, for M1, there are 52 muscle synergies extracted from 10 participants, among them, 19 synergy structures are identified as intra-mode shared synergy structure, accounting for 36.5%. However, 37 recruitment curves of the 52 muscle synergies are identified as intra-mode shared recruitment curve, accounting for 71.2%. Similarly, in modes M2 ~ M8, the proportions of the intra-mode shared synergy structures are 41.2, 67.3, 68.8, 66, 68, 56 and 60% respectively, however, the proportions of intra-mode recruitment curves are 76.5, 83.7, 91.7, 88.7, 94, 84 and 92%, respectively. Additionally, the intra-mode shared recruitment curves have higher similarity (0.87 ± 0.05) than the intra-mode shared synergy structure (0.83 ± 0.02) among participants.

Secondly, through the analysis of the extraction results of inter-mode shared synergies, it can be found that CNS mainly realizes single-limb swing crawling modes M3 ~ M8 by recruiting the 4 inter-mode shared synergy structures whose main function is to complete the swing of each limb. As shown in Figure 7, the 4 inter-mode shared recruitment curves are extracted in the eight crawling modes. Pattern 1 to Pattern 4 represent each quarter of the crawling cycle from the beginning to the end. Since the M3 ~ M8 is defined as the limbs swinging successively and the time duration of each limb swinging is exactly 1/4 crawling cycle, Pattern 1 to Pattern 4 actually correspond to the swing phases of the four limbs. Taking M3 as example, the order of limbs swing is right hand→ left knee→ right knee→ left hand as shown in Table 1. It can be observed in Figure 7 that from Pattern 1 to Pattern 4, the recruitment curves are successively C1, C4, C2, and C3, namely the recruitment sequence of the 4 inter-shared synergy structures is W1, W4, W2, and W3 in a crawling cycle. Table 4 summarizes the recruitment order of the intra-shared synergy structures under the eight crawling modes. It can be found that for M3 ~ M8, W1, W2, W3, and W4 are recruited in different order according to the order of limbs swing.

**Table 4:** The recruited sequence of the intra-mode shared synergy structures.

	Pattern 1	Pattern 2	Pattern 3	Pattern 4
M1		W1, W5		W6, W7
M2		W1, W8		W3
M3	W1	W4	W2	W3
M4	W1	W2	W4	W3
M5	W4	W1	W2	W3
M6	W4	W2	W1	W3
M7	W2	W1	W4	W3
M8	W2	W4	W1	W3

In each synergy structure, the degree of activation of the muscle is scaled from 0 to 1, and the muscles activated more than 0.5 are defined as high level of activation. As shown in Figures 4, 6 inter-mode shared synergy structures W1 ~ W4 are extracted from the eight inter-limb coordination modes. In particular, W1 ~ W4 are shared by all the 6 single-limb swing modes M3 ~ M8. For W1, the muscles 1-TR, 4-TB, 16-TR, 18-BB, 20-BR, 21-ECR, and 23-LD are highly activated; for W2, the muscles 8-LD, 9-RF,10-VL, 11-VM, 17-AD, 19-TB, 22-FCR, 27-SA, 28-AL, 29-BF, and 30-SE are highly activated; for W3, the muscles 1-TR, 3-BB, 5-BR, 6-ECR, 8-LD, 15-SE, 16-TR, 17-AD, and 19-TB are highly activated; for W4, the muscles 4-TB, 7-FCR, 12-SA, 13-AL, 14-BF, 15-SE, 24-RF, 25-VL, 26-VM, and 27-SA are highly activated. Since the muscles TR, BB, BR, ECR and LD can provide the impetus for the hand to swing forward, W1 and W3 are responsible for the swing of the right hand and the swing of the left hand, respectively. The muscles SA, AL, BF, and SE can provide the impetus for the leg to swing forward, and the muscles RF, VL, and VM on the other side leg are to keep the body stable, so W2 and W4 are responsible for the swing of the right leg and the swing of the left leg, respectively.

Thirdly, through the analysis of the intra-mode and inter-mode shared synergies, it can be found that CNS realizes the two-limb swing modes M1 and M2 by recruiting specific intra-mode shared synergy structures. As shown in Figure 6, only one inter-mode shared synergy structure W1 is extracted in M1 and two inter-mode shared synergy structures W1 and W3 are extracted in M2. W5, W6, and W7 are the specific synergy structures that only appear in M1, and W8 is the specific synergy structure that only appears in M2. In the meanwhile, unlike the single-limb swing modes, which recruit the four inter-mode shared synergies in turn, the two-limb swing modes sometimes recruit two synergies simultaneously. As shown in Figure 7, in crawling mode M1, W1 and W5 are recruited together, and W6 and W7 are recruited together. In crawling mode M2, W1 and W8 are recruited together.

In crawling mode M1, W1, and W5 are recruited together to complete the swing of the right hand and the right leg simultaneously, and W6 and W7 are recruited together to complete the swing of the left hand and the left leg. The muscles 20-BR, 21-ECR, 23-LD, 27-SA, 28-AL of W1 and the muscles 18-BB, 20-BR, 21-ECR, 23-LD, 30-SE of W5 are high activated to provide the impetus for the right hand and right leg to swing forward; the muscles 1-TR, 2-AD, 3-BB, 4-TB of W1 and the muscles 9-RF, 10-VL, 11-VM, 14-BF, 15-SE of W5 are highly activated to keep the body stable. The muscles 5-BR, 6-ECR, 8-LD, 12-SA, 13-AL of W6 and the muscles 3-BB, 5-BR, 6-FCR, 8-LD, 15-SE of W7 are high activated to provide the impetus for the left hand and left leg to swing forward; the muscles 24-RF, 25-VL, 26-VM, 29-BF, 30-SE of W6 and the muscles 16-TR, 17-AD, 18-BB, 19-TB of W7 are high activated to keep the body stable.

In crawling mode M2, W1, and W8 are recruited together to complete the swing of the right hand and the left leg simultaneously. The muscles 14-BF, 15-SE, 20-BR, 21-ECR, 23-LD of W1 and the muscles 12-SA, 13-AL, 17-AD, 19-TB, 22-FCR of W8 are highly activated to provide the impetus for the right hand and left leg to swing forward, and the muscles 4-TB, 24-RF, 25-VL, 26-VM of W1, and the muscles 1-TR, 2-AD, 3-BB, 4-TB, 7-FCR of W8 are high activated to keep the body stable. For W3, the muscles 3-BB, 5-BR, 6-ECR, 8-LD, 29-BF, and 30-SE are highly activated provide the impetus for the left hand and right leg to swing forward; The muscles 9-RF, 10-VL, 11-VM, 19-TB are highly activated to keep the body stable.

## 4. Discussion

This study carries out muscle synergy extraction and analysis on human crawling movement with eight specific inter-limb coordination modes. The research results have the following significance for people to further understand the neuromuscular control mechanism of crawling movement.

### 4.1. Verification of the muscle synergy hypothesis in crawling movement

Numerous studies on human locomotion, such as reaching (d’Avella et al., 2006), cycling (De Marchis et al., 2013), bench press (Kristiansen et al., 2016), walking (Shuman et al., 2016; Taborri et al., 2018), and rowing (Turpin et al., 2011), have demonstrated that muscle synergies are robust and have similar control mechanism under various experimental conditions, including intra-day and inter-day, and intra-participant and inter-participant (Torres-Oviedo et al., 2006; Turpin et al., 2011; De Marchis et al., 2013; Kristiansen et al., 2016; Shuman et al., 2016; Taborri et al., 2017; Santos et al., 2020). Santos et al. evaluated the intra-and inter-day reliability of muscle synergy of a strength training complex task (the power clean). They found that high intra-day similarity values were shown in synergy structure matrix, synergy recruitment curves, and individual electromyographic profiles. Inter-day muscle synergy structures had moderate similarity, while the synergy recruitment curves were strongly related (Santos et al., 2020). For walking gait, Shuman et al. investigated the repeatability of muscle synergies between days for both typically developing children and children with cerebral palsy, and found there was no significant inter-day difference in muscle synergies of the two children groups (Shuman et al., 2016). In another study on walking, Taborri et al. quantified the intra-participant and inter-participant variability of muscle synergy, and found that both the intra-participant and inter-participant variability were negligible. Based on the intra-limb muscle coordination analysis, high structural similarity of muscle synergies was found among participants (Taborri et al., 2018).

As mentioned in the Introduction, the works of Chen et al. (2017), Gao et al. (2018), Xiong et al. (2018), and Li et al. (2019) showed that the muscle synergy hypothesis also had been verified in human crawling. However, the existing researches mainly involved two inter-limb coordination modes, namely pace-like mode and trot-like mode, and the research results were relatively preliminary. The research results of this paper provided new evidence for the muscle synergy hypothesis from the perspective of crawling movement. 4 ~ 7 muscle synergies were extracted from each participant in each inter-limb coordination mode, which demonstrated that CNS controls the inter-limb coordination modes in human crawling by recruiting a certain amount of muscle synergies, rather than a single muscle. The above result supports the already known body of knowledge that synergy exists in the sequential stimulation of the muscles of the limbs so that they finally as a team completes a movement. Further, similar to other human locomotion such as walking etc., the muscle synergies were found to be shared among participants and crawling modes. When different participants crawled with the same inter-limb coordination mode, 3 ~ 4 intra-mode shared synergies were extracted. Through the analysis of the intra-mode shared synergies, we found that the control of CNS on human crawling had better commonality in the aspect of synergy recruitment pattern than synergy structure. That is to say, when different participants completed the same crawling mode, they shared more temporal features.

In the study on the intra-limb muscle coordination of adult crawling movements, Chen et al. found that two synergies could be extracted from each upper limb to represent the swing phase and stance phase, respectively (Chen et al., 2017). The authors claimed that, a two-level central pattern generator (CPG) model (Brown, 1911), which consisted of a half-center rhythm generator (RG) and a pattern formation (PF) circuit, can be adopted to explain the above results. In this study, four inter-mode shared synergies, which are related to the swing of the four limbs, were extracted. The results of the inter-limb coordination analysis conformed to CPG model well. The PF circuit can be represented by synergy structures, and the RG can be represented by recruitment curves. RG mediated the recruitment of the swing phase related synergies to control one certain limb, four limbs were recruited one by one to finish the crawling task in modes M3 ~ M8, and two limbs were recruited together to finish the crawling task in modes M1 and M2.

In addition, as an already known body of knowledge, crawling is a milestone in a developing human from an infant. When the neural stimulation occurs from the motor cortex, concerted contraction of the regional muscles happens so that the supporting system of the body first has four points of contact with the ground and later has two points of contact. In this study, by analyzing intra-mode shared synergies among participants and inter-mode shared synergies among the eight inter-limb coordination modes, the CNS is found to realize single-limb swing crawling modes by recruiting the four inter-mode shared synergy structures related to the swing function of each limb in different orders, and realize the two-limb swing crawling modes by recruiting synchronously two intra-mode shared synergy structures. Therefore, the realization of single-limb swing crawling modes M3 ~ M8 is easier than that of two-limb swing crawling modes M1 ~ M2. It may be the reason why infants first have four points of contact with ground and later have two points of contact.

### 4.2. The possible explanation for the choice of inter-limb coordination mode during human crawling

Crawling is the first mobility for most human beings (Adolph et al., 1998) and it plays a vital role in human growth and development. Various inter-limb coordination modes are available when human beings crawl (Adolph et al., 1998). As mentioned in Introduction, the inter-limb coordination modes of human beings can be classified according to the sequence and relative timing of limb movements, and they can be mainly divided into pace-like mode, in which the ipsilateral limbs move together; trot-like mode, in which the diagonal limbs move together; and no-limb-pairing mode, in which all limbs move at regular intervals. Ma et al. found that human adults preferred to crawl in trot-like gait and no-limb-pairing gait at low speed, and preferred to crawl in trot-like gait and pace-like gait rather than no-limb-pairing gait at high speed (≥ 2 km/h) (Ma et al., 2017). Chen et al. also found that human adults would like to choose no-limb-pairing gait at low speed and pace-like gait or trot-like gait at high speed (Chen et al., 2017). Righetti et al. called the trot-like gait as “the standard crawling gait” and claimed that although there were very different strategies for infants crawling, i.e., the belly touching ground or not, using four limbs or three limbs, with different types of limb coordination, the trot-like gait was the most common gait (Righetti et al., 2015). Freedland et al. found that infants choose trot-like gait in their second week on hands-and-knees crawling (Freedland and Bertenthal, 1994). Cole et al. found that the trot-like gait was the predominant gait not only in hands-and-knees crawling but also in hands-and-feet crawling (Cole et al., 2019). Patrick et al. claimed that trot-like gait was the predominant gait of infants, and the pace-like gait was never observed (Patrick et al., 2012).

In summary, existing studies show that trot-like is the most widely used inter-limb coordination mode in crawling, whether in adults or infants, at low or high speeds. No-limb-pairing gait only occasionally occurs in adults at low speed crawling, and pace-like mode only occasionally occurs in adults at high speed crawling. However, the reason why human beings choose the specific inter-limb coordination modes during crawling remains to be explored. Based on the results of the muscle synergy analysis of human crawling movement in this paper, we attempt to explain the possible reasons for the choice of inter-limb coordination modes in human crawling as below:

1. No-limb-pairing is a mode of single-limb swing or three-limb support, and trot-like and pace-like are two-limb swing/support modes. In this study, through the analysis of the shared muscle synergies, we found that CNS mainly realizes no-limb-pairing crawling modes M3 ~ M8 by recruiting the 4 inter-mode shared synergy structures related to the swing of the four limbs in turn, however, controls the pace-like crawling mode M1 and trot-like crawling mode M2 by recruiting specific intra-mode shared synergy structures. Relatively speaking, the three-limb support provides better stability than the two-limb support, at the same time, it is easier to control the swing of one limb alone than to control the swing of two limbs simultaneously, which maybe the reason why some adults occasionally choose no-limb-pairing gait at low speed crawling. However, the swing of the four limbs in turn makes it difficult to improve the crawling speed. The case that recruiting intra-mode shared synergies simultaneously seems to be more beneficial to improving the stride frequency than recruiting inter-limb shared synergies in turn, and it may be the reason why adults would like to adopt trot-like mode or pace-like mode at high speed.
2. Trot-like is the two-limb swing/support mode using contralateral limbs. In the crawling process, the contralateral limbs swing or support at the same time, which is easy to maintain balance at both high and low speed. Vitali et al. (2019) found that trot-like gait can exhibit superior limb coordination, and can produce faster crawl speeds and shorter crawl stride times. Alexander (1992) claimed that although many inter-limb coordination modes would appear during infants crawling, the trot-like gait was most biomechanically efficient and stable. In this study, the hands-knees crawling movement is the research object. Relatively speaking, the upper limbs play a more dominant role in the crawling process. The results of Figure 6 show that the trot-like mode M2 and no-limb-pairing mode M3 ~ M8 share synergy structures W1 and W3, which correspond to swing functions of right hand and left hand, respectively. Meanwhile, it can be observed from Figure 7 that W1 and W3 play a decisive role in the Pattern 2 and the Pattern 4, respectively. The above results imply that trot-like mode can provide similar stability comparing to no-limb-pairing mode. At the same time, as mentioned above, the two-limb swing mode has advantages in improving the crawling speed than the single-limb swing mode. Therefore, the trot-like mode has become the crawling mode that humans are most willing to choose.
3. Pace-like is the two-limb swing/support mode using ipsilateral limbs. During crawling, the ipsilateral limbs swing or support at the same time, which is not easy to maintain balance. Patrick et al. (2012) pointed out that no-limb-pairing gait and trot-like gait could offer higher stability than pace-like gait, and the pace-like gait seemly required a more mature nervous system (Patrick et al., 2012). The results of this study (Figure 6) show that the pace-like gait M1 and no-limb-pairing gait M3 ~ M8 only share the synergy structure W1 corresponding to the swing function of right hand (and the similarity is low). Meanwhile, it can be observed from Figure 7 that W1 only plays a decisive role in the Pattern 2. The above results imply that the stability of pace-like gait is lower than the trot-like gait and no-limb-pairing gait, which supports the viewpoint of Patrick et al. As for why some adults occasionally choose pace-like gait at high speed, we speculated that the reason is the reduced need for stability at high speed.

### 4.3. Limitations

This study has some limitations and its future work directions are as follows: at first, although hands-and-knees crawling is the posture mainly adopted by human beings, the muscle synergy analysis needs to expand to other crawling postures, such as hands-feet, scooting, creeping on the belly; Secondly, it is necessary to expand our work to different age groups such as infants, children and the elderly; Thirdly, the methods and results of this study whether can be applied to the clinical analysis is needed to be demonstrated.

## 5. Conclusion

Different from the relevant studies, which focus on muscle synergy analysis of autonomously selected inter-limb coordination modes at self-selected crawling speed (Patrick et al., 2009, 2012; Righetti et al., 2015; Xiong et al., 2018), the novelty of the crawling research carried out in this paper is that it expands the research object from the three typical modes to eight modes. To the best of our knowledge, this is the first work to conduct muscle synergy analysis on the eight inter-limb coordination modes during crawling movements. The main contribution of this study is that it reveals the following neuromuscular control properties of human crawling, including: (1) CNS controls the crawling movement by recruiting a certain amount of muscle synergies, rather than a single muscle, and different participants share more temporal features in recruiting muscle synergies when they complete the same crawling mode; (2) CNS mainly realizes single-limb swing crawling modes by recruiting the 4 inter-mode shared synergy structures related to the swing phase of each limb in different orders, and realizes the two-limb swing crawling modes by recruiting two specific intra-mode shared synergy structures at the same time. Based on the results of the muscle synergy analysis on human crawling movement, another contribution of this study is to try to explain the possible reasons for the choice of inter-limb coordination modes during crawling. The research results of this paper contribute to the in-depth understanding of the neural control mechanism of CNS on human crawling.

## Data availability statement

The raw data supporting the conclusions of this article will be made available by the authors, without undue reservation.

## Ethics statement

The studies involving human participants were reviewed and approved by The Ethics Review Committee of Anhui Medical University approves this study (No. PJ 2014-08-04). The patients/participants provided their written informed consent to participate in this study.

## Author contributions

CL and XiC designed this study and drafted the manuscript. CL conducted the experiments. CL, XiC, XZ, XuC, and DW performed data analyses and interpretations, and conceived the experiments. XuC and DW substantially revised the manuscript. All authors read and approved the final version of the manuscript.

## Funding

This work was supported by the National Natural Science Foundation of China under Grant nos. 82272113 and 61671417.

## Conflict of interest

The authors declare that the research was conducted in the absence of any commercial or financial relationships that could be construed as a potential conflict of interest.

## Publisher’s note

All claims expressed in this article are solely those of the authors and do not necessarily represent those of their affiliated organizations, or those of the publisher, the editors and the reviewers. Any product that may be evaluated in this article, or claim that may be made by its manufacturer, is not guaranteed or endorsed by the publisher.

## References

[ref1] Adolph K. E. Vereijken B. Denny M. A. (1998). Learning to crawl. Child Dev. 69, 1299–1312. doi: 10.2307/1132267 9839417

[ref2] Alexander R. M., Exploring biomechanics New York: WH Freeman (1992).

[ref3] Bizzi E. Cheung V. C. d’Avella A. Saltiel P. Tresch M. (2008). Combining modules for movement. Brain Res. Rev. 57, 125–133. doi: 10.1016/j.brainresrev.2007.08.004, PMID: 18029291 PMC4295773

[ref4] Brown T. G. (1911). The intrinsic factors in the act of progression in the mammal. Proceedings of the Royal Society of London Series B, containing papers of a biological character 84, 308–319.

[ref5] Chen X. Niu X. Wu D. Yu Y. Zhang X. (2017). Investigation of the intra-and inter-limb muscle coordination of hands-and-knees crawling in human adults by means of muscle synergy analysis. Entropy 19:229. doi: 10.3390/e19050229

[ref6] Cheung V. C. Turolla A. Agostini M. Silvoni S. Bennis C. Kasi P.. (2012). Muscle synergy patterns as physiological markers of motor cortical damage. Proc. Natl. Acad. Sci. U. S. A. 109, 14652–14656. doi: 10.1073/pnas.1212056109, PMID: 22908288 PMC3437897

[ref7] Clark D. J. Ting L. H. Zajac F. E. Neptune R. R. Kautz S. A. (2010). Merging of healthy motor modules predicts reduced locomotor performance and muscle coordination complexity post-stroke. J. Neurophysiol. 103, 844–857. doi: 10.1152/jn.00825.2009, PMID: 20007501 PMC2822696

[ref8] Cole W. G. Vereijken B. Young J. W. Robinson S. R. Adolph K. E. (2019). Use it or lose it? Effects of age, experience, and disuse on crawling. Dev. Psychobiol. 61, 29–42. doi: 10.1002/dev.21802, PMID: 30447002 PMC6345180

[ref9] d’Avella A. Portone A. Fernandez L. Lacquaniti F. (2006). Control of fast-reaching movements by muscle synergy combinations. J. Neurosci. 26, 7791–7810. doi: 10.1523/JNEUROSCI.0830-06.2006, PMID: 16870725 PMC6674215

[ref10] d’Avella A. Portone A. Lacquaniti F. (2011). Superposition and modulation of muscle synergies for reaching in response to a change in target location. J. Neurophysiol. 106, 2796–2812. doi: 10.1152/jn.00675.2010, PMID: 21880939

[ref11] d’Avella A. Saltiel P. Bizzi E. (2003). Combinations of muscle synergies in the construction of a natural motor behavior. Nat. Neurosci. 6, 300–308. doi: 10.1038/nn1010, PMID: 12563264

[ref12] De Marchis C. Schmid M. Bibbo D. Bernabucci I. Conforto S. (2013). Inter-individual variability of forces and modular muscle coordination in cycling: a study on untrained subjects. Hum. Mov. Sci. 32, 1480–1494. doi: 10.1016/j.humov.2013.07.018, PMID: 24060224

[ref13] Flash T. Hochner B. (2005). Motor primitives in vertebrates and invertebrates. Curr. Opin. Neurobiol. 15, 660–666. doi: 10.1016/j.conb.2005.10.011, PMID: 16275056

[ref14] Freedland R. L. Bertenthal B. I. (1994). Developmental changes in interlimb coordination: transition to hands-and-knees crawling. Psychol. Sci. 5, 26–32. doi: 10.1111/j.1467-9280.1994.tb00609.x

[ref15] Gao Z. Chen L. Xiong Q. Xiao N. Jiang W. Liu Y.. (2018). Degraded synergistic recruitment of sEMG oscillations for cerebral palsy infants crawling. Front. Neurol. 9:e00760. doi: 10.3389/fneur.2018.00760 PMC6153367 30279674

[ref16] Hermens H. J. Freriks B. Disselhorst-Klug C. Rau G. (2000). Development of recommendations for SEMG sensors and sensor placement procedures. J. Electromyogr. Kinesiol. 10, 361–374. doi: 10.1016/S1050-6411(00)00027-4, PMID: 11018445

[ref17] Hyvärinen A. Oja E. (2000). Independent component analysis: algorithms and applications. Neural Netw. 13, 411–430. doi: 10.1016/S0893-6080(00)00026-5, PMID: 10946390

[ref18] Kieliba P. Tropea P. Pirondini E. Coscia M. Micera S. Artoni F. (2018). How are muscle synergies affected by electromyography pre-processing? IEEE Trans. Neural Syst. Rehabil. Eng. 26, 882–893. doi: 10.1109/TNSRE.2018.2810859, PMID: 29641393

[ref19] Kretch K. S. Franchak J. M. Adolph K. E. (2014). Crawling and walking infants see the world differently. Child Dev. 85, 1503–1518. doi: 10.1111/cdev.12206, PMID: 24341362 PMC4059790

[ref20] Kristiansen M. Samani A. Madeleine P. Hansen E. A. (2016). Muscle synergies during bench press are reliable across days. J. Electromyogr. Kinesiol. 30, 81–88. doi: 10.1016/j.jelekin.2016.06.004, PMID: 27323305

[ref21] Kurtzer I. Pruszynski J. A. Herter T. M. Scott S. H. (2006). Primate upper limb muscles exhibit activity patterns that differ from their anatomical action during a postural task. J. Neurophysiol. 95, 493–504. doi: 10.1152/jn.00706.2005, PMID: 16251262

[ref22] Kutch J. J. Kuo A. D. Bloch A. M. Rymer W. Z. (2008). Endpoint force fluctuations reveal flexible rather than synergistic patterns of muscle cooperation. J. Neurophysiol. 100, 2455–2471. doi: 10.1152/jn.90274.2008, PMID: 18799603 PMC2585402

[ref23] Lee D. D. Seung H. S. (1999). Learning the parts of objects by non-negative matrix factorization. Nature 401, 788–791. doi: 10.1038/44565, PMID: 10548103

[ref24] Li T. Chen X. Cao S. Zhang X. Chen X. (2019). Human hands-and-knees crawling movement analysis based on time-varying synergy and synchronous synergy theories. Math. Biosci. Eng. 16, 2492–2513. doi: 10.3934/mbe.2019125, PMID: 31137224

[ref25] Ma S. Chen X. Cao S. Yu Y. Zhang X. (2017). Investigation on inter-limb coordination and motion stability, intensity and complexity of trunk and limbs during hands-knees crawling in human adults. Sensors 17:692. doi: 10.3390/s17040692, PMID: 28350324 PMC5421652

[ref26] McEwan M. H. Dihoff R. E. Brosvic G. M. (1991). Early infant crawling experience is reflected in later motor skill development. Percept. Mot. Skills 72, 75–79. doi: 10.2466/pms.1991.72.1.75, PMID: 2038537

[ref27] Murtagh F. Contreras P. (2017). Algorithms for hierarchical clustering: an overview, II. Wiley interdisciplinary reviews. Data Min. Know. Discov. 7:e1219. doi: 10.1002/widm.1219

[ref28] Patrick S. K. Noah J. A. Yang J. F. (2009). Interlimb coordination in human crawling reveals similarities in development and neural control with quadrupeds. J. Neurophysiol. 101, 603–613. doi: 10.1152/jn.91125.2008, PMID: 19036860 PMC2657078

[ref29] Patrick S. K. Noah J. A. Yang J. F. (2012). Developmental constraints of quadrupedal coordination across crawling styles in human infants. J. Neurophysiol. 107, 3050–3061. doi: 10.1152/jn.00029.2012, PMID: 22402655 PMC3378364

[ref30] Rabbi M. F. Pizzolato C. Lloyd D. G. Carty C. P. Devaprakash D. Diamond L. E. (2020). Non-negative matrix factorisation is the most appropriate method for extraction of muscle synergies in walking and running. Sci. Rep. 10, 1–11. doi: 10.1038/s41598-020-65257-w 32427881 PMC7237673

[ref31] Ranganathan R. Krishnan C. Dhaher Y. Y. Rymer W. Z. (2016). Learning new gait patterns: exploratory muscle activity during motor learning is not predicted by motor modules. J. Biomech. 49, 718–725. doi: 10.1016/j.jbiomech.2016.02.006, PMID: 26916510 PMC5796520

[ref32] Righetti L. Nylén A. Rosander K. Ijspeert A. J. (2015). Kinematic and gait similarities between crawling human infants and other quadruped mammals. Front. Neurol. 6:17. doi: 10.3389/fneur.2015.00017 25709597 PMC4321575

[ref33] Saltiel P. Wyler-Duda K. D’Avella A. Tresch M. C. Bizzi E. (2001). Muscle synergies encoded within the spinal cord: evidence from focal intraspinal NMDA iontophoresis in the frog. J. Neurophysiol. 85, 605–619. doi: 10.1152/jn.2001.85.2.605, PMID: 11160497

[ref34] Santos P. D. Vaz J. R. Correia P. F. Valamatos M. J. Veloso A. P. Pezarat-Correia P. (2020). Muscle synergies reliability in the power clean exercise. J. Funct. Morphol. Kinesiol. 5:75. doi: 10.3390/jfmk5040075, PMID: 33467290 PMC7739416

[ref35] Shuman B. Goudriaan M. Bar-On L. Schwartz M. H. Desloovere K. Steele K. M. (2016). Repeatability of muscle synergies within and between days for typically developing children and children with cerebral palsy. Gait Posture 45, 127–132. doi: 10.1016/j.gaitpost.2016.01.011, PMID: 26979894

[ref36] Taborri J. Agostini V. Artemiadis P. K. Ghislieri M. Jacobs D. A. Roh J.. (2018). Feasibility of muscle synergy outcomes in clinics, robotics, and sports: a systematic review. Appl. Bionics Biomech. 2018, 1–19. doi: 10.1155/2018/3934698, PMID: 29808098 PMC5902115

[ref37] Taborri J. Palermo E. Masiello D. Rossi S., Factorization of EMG via muscle synergies in walking task: evaluation of intra-subject and inter-subject variability (2017). IEEE international instrumentation and measurement technology conference (I2MTC), IEEE. doi: 10.1109/I2MTC.2017.7969775

[ref38] Tang L. Chen X. Cao S. Wu D. Zhao G. Zhang X. (2017). Assessment of upper limb motor dysfunction for children with cerebral palsy based on muscle synergy analysis. Front. Hum. Neurosci. 11:130. doi: 10.3389/fnhum.2017.00130 28386223 PMC5362624

[ref39] Tang L. Li F. Cao S. Zhang X. Wu D. Chen X. (2015). Muscle synergy analysis in children with cerebral palsy. J. Neural Eng. 12:046017. doi: 10.1088/1741-2560/12/4/046017 26061115

[ref40] Ting L. H. Chvatal S. A. (2010). Decomposing muscle activity in motor tasks. Motor Control, 102–138. doi: 10.1093/acprof:oso/9780195395273.003.0005

[ref41] Ting L. H. Macpherson J. M. (2005). A limited set of muscle synergies for force control during a postural task. J. Neurophysiol. 93, 609–613. doi: 10.1152/jn.00681.2004, PMID: 15342720

[ref42] Torres-Oviedo G. Macpherson J. M. Ting L. H. (2006). Muscle synergy organization is robust across a variety of postural perturbations. J. Neurophysiol. 96, 1530–1546. doi: 10.1152/jn.00810.2005, PMID: 16775203

[ref43] Tresch M. C. Jarc A. (2009). The case for and against muscle synergies. Curr. Opin. Neurobiol. 19, 601–607. doi: 10.1016/j.conb.2009.09.002, PMID: 19828310 PMC2818278

[ref44] Tresch M. C. Saltiel P. d’Avella A. Bizzi E. (2002). Coordination and localization in spinal motor systems. Brain Res. Rev. 40, 66–79. doi: 10.1016/S0165-0173(02)00189-3 12589907

[ref45] Turpin N. A. Guével A. Durand S. Hug F. (2011). No evidence of expertise-related changes in muscle synergies during rowing. J. Electromyogr. Kinesiol. 21, 1030–1040. doi: 10.1016/j.jelekin.2011.07.013, PMID: 21856171

[ref46] Valero-Cuevas F. J. Venkadesan M. Todorov E. (2009).Structured variability of muscle activations supports the minimal interventionprinciple of motor control. J. Neurophysiol. 102, 59–68. doi: 10.1152/jn.90324.2008, PMID: 19369362 PMC2712269

[ref47] Vitali R. V. Cain S. M. Davidson S. P. Perkins N. C. (2019). Human crawling performance and technique revealed by inertial measurement units. J. Biomech. 84, 121–128. doi: 10.1016/j.jbiomech.2018.12.030, PMID: 30638720

[ref48] Wojtara T. Alnajjar F. Shimoda S. Kimura H. (2014). Muscle synergy stability and human balance maintenance. J. Neuroeng. Rehabil. 11, 1–9. doi: 10.1186/1743-0003-11-129 25174281 PMC4161771

[ref49] Xiong Q. L. Wu X. Y. Liu Y. Zhang C. X. Hou W. S. (2021). Measurement and analysis of human infant crawling for rehabilitation: a narrative review. Front. Neurol. 12:e731374. doi: 10.3389/fneur.2021.731374 PMC8544808 34707557

[ref50] Xiong Q. L. Wu X. Y. Yao J. Sukal-Moulton T. Xiao N. Chen L.. (2018). Inter-limb muscle synergies and kinematic analysis of hands-and-knees crawling in typically developing infants and infants with developmental delay. Front. Neurol. 9:e00869. doi: 10.3389/fneur.2018.00869 PMC6198063 30386289

